# Log-Cubic Method for Generation of Soil Particle Size Distribution Curve

**DOI:** 10.1155/2013/579460

**Published:** 2013-05-20

**Authors:** Songhao Shang

**Affiliations:** ^1^State Key Laboratory of Hydroscience and Engineering, Tsinghua University, Beijing 100084, China; ^2^Department of Hydraulic Engineering, Tsinghua University, Beijing 100084, China

## Abstract

Particle size distribution (PSD) is a fundamental physical property of soils. Traditionally, the PSD curve was generated by hand from limited data of particle size analysis, which is subjective and may lead to significant uncertainty in the freehand PSD curve and graphically estimated cumulative particle percentages. To overcome these problems, a log-cubic method was proposed for the generation of PSD curve based on a monotone piecewise cubic interpolation method. The log-cubic method and commonly used log-linear and log-spline methods were evaluated by the leave-one-out cross-validation method for 394 soil samples extracted from UNSODA database. Mean error and root mean square error of the cross-validation show that the log-cubic method outperforms two other methods. What is more important, PSD curve generated by the log-cubic method meets essential requirements of a PSD curve, that is, passing through all measured data and being both smooth and monotone. The proposed log-cubic method provides an objective and reliable way to generate a PSD curve from limited soil particle analysis data. This method and the generated PSD curve can be used in the conversion of different soil texture schemes, assessment of grading pattern, and estimation of soil hydraulic parameters and erodibility factor.

## 1. Introduction

Particle size distribution (PSD) is a fundamental physical property of soils, which can be described by the PSD curve of cumulative particle percentage versus logarithm of particle size. The PSD curve provides detailed information about the soil, such as grading pattern and the sand, silt, and clay fractions to determine the soil textural classes [1]. It is also useful for the conversion of different soil texture schemes [2]. What is more, these textural fractions are more readily available from particle size analysis or existing soil database, so they are usually taken as main inputs to estimate other soil properties difficult to obtain, such as hydraulic properties [3–5] and soil erodibility factor [6, 7].

In the practice of particle size analysis, only limited data of cumulative particle percentage versus particle size are available. Traditionally, these limited data were plotted on semilogarithmic coordinates, and then these points were smoothly connected by hand to generate a smooth and monotone PSD curve. After the generation of a PSD curve, cumulative percentage at unmeasured size and characteristic particle size corresponding to specified cumulative percentage can be estimated from the curve graphically. However, the previous processes are subjective, which may lead to significant uncertainty in the freehand PSD curve and graphically estimated cumulative particle percentage and characteristic particle size [8]. To overcome the subjectivity of freehand PSD curve, regression and interpolation methods and similarity procedure had been used to estimate cumulative particle percentages at unmeasured particle sizes.

Regression method was used to fit the PSD curve with various empirical formulae [9, 10], which had been evaluated with measured data from different part of the world [11–13]. These empirical PSD curves can represent the trend of cumulative percentage varying with particle size and can be used in the estimation of soil hydraulic properties [4, 14]. However, the fitted empirical curves may not be flexible enough to depict PSD of diverse soil types. Besides, they usually do not pass through measured data, which is not in accord with the essential requirement of a PSD curve.

The similarity procedure to estimate cumulative percentage at specified unmeasured size of a soil sample is based on the similarity of PSD between soil under consideration and an external reference data set [15], on condition that data corresponding to the specified particle size were available from the reference data set. Therefore, this procedure is not suitable for the generation of a continuous PSD curve. Besides, because a large external reference data set is required to find soils with similar PSDs, this procedure was not often used due to the lack of appropriate reference data set.

Interpolation method was also used to approximate the PSD with a function passing through measured data, which is similar to artificially plotted PSD curve. Main methods for the interpolation of PSD curves include the log-linear interpolation [15, 16] and the cubic spline [8]. The log-linear interpolation curve can ensure the monotonicity of the PSD curve, but it is not smooth. The cubic spline is smooth, but it is monotone only in specified conditions of measured data [17]. In some cases, cubic spline may produce impractical results, which can be overcome by modifying impractical results with regression analysis results or dividing the whole range of particle size into two segments and constructing a spline for each segment [8]. However, these modifications may be only applicable to specific conditions. It is still necessary to find a simple and reliable method to generate a PSD curve from limited data.

The main purpose of this study was to propose a log-cubic method to generate the PSD curve from limited soil particle analysis data, which is based on a monotone piecewise cubic interpolation method [17]. This method was evaluated with the leave-one-out cross-validation method for 394 soil samples extracted from UNSODA database [18].

## 2. Materials and Methods

### 2.1. The Log-Cubic Method

Generally, cumulative particle percentages are available for limited sizes from soil particle size analysis. Suppose that *n* points, (*d*_*i*_, *P*_*i*_), *i* = 1, 2, …, *n*, were available, where *P*_*i*_ is the cumulative particle percentage corresponding to particle size *d*_*i*_. Since a PSD curve is both smooth and monotone, it can be approximated with the monotone piecewise cubic interpolation method [17]. 

Considering that the particle size covers several orders of magnitude and the PSD curve is plotted on semilogarithmic coordinates, the logarithm of particle size (ln⁡*d*) was used in the interpolation. Similar to the log-linear method, the proposed method was named as the log-cubic method. The log-cubic interpolation function, *P*_*c*_(*d*), is composed of *n* − 1 cubic polynomial segments defined in particle size intervals [*d*_*i*_, *d*_*i*+1_], *i* = 1, 2, …, *n* − 1, which can be described by (1) and (2):

(1) Pc(d)=Pci(d), di≤d≤di+1, i=1,2,…,n−1,

(2) Pci(d)=Pi+13hs2−2s3h3+Pih3−3hs2+2s3h3+fi+1s2(s−h)h2+fis(s−h)2h2, i=1,2,…,n−1,

where *P*_*ci*_(*d*) is the segment of *P*_*c*_(*d*) for particle size interval of [*d*_*i*_, *d*_*i*+1_], *h* = ln⁡*d*_*i*+1_ − ln⁡*d*_*i*_, *s* = ln⁡*d* − ln⁡*d*_*i*_, and *f*_*i*_ and *f*_*i*+1_ denote the slope of *P*_*c*_(*d*) at knots *d*_*i*_ and *d*_*i*+1_, respectively. The slope at a knot can be estimated from the lengths and the first divided differences of two adjacent intervals [17, 19]. This interpolation method has been used in several fields of soil and agricultural sciences [20–22].

The log-cubic interpolation function defined in (1) passes through all measured points and is both smooth and monotone, which meets essential requirements of a PSD curve.

For comparison, commonly used log-linear [15, 16] and log-spline [8] methods were also used. The log-linear interpolation function for PSD can be described by (3):

(3) $$\begin{array}{c} P_{l}\left(d\right)=P_{li}\left(d\right),\;d_{i}\leq d\leq d_{i+1},\;\;i=1,2,\ldots ,n-1,\\ P_{li}\left(d\right)=P_{i}\frac{h-s}{h}+P_{i+1}\frac{s}{h},\;i=1,2,\ldots ,n-1, \end{array}$$

where *P*_*li*_(*d*) is the segment of log-linear interpolation function, *P*_*l*_(*d*), for *d*_*i*_ ≤ *d* ≤ *d*_*i*+1_. 

The log-spline method for PSD is based on the cubic spline interpolation of *P* versus ln⁡*d*, which can be described by (4):

(4) Ps(d)=Psi(d), di≤d≤di+1,  i=1,2,…,n−1,Psi(d)=Mi(h−s)36h+Mi+1s36h+(Pih−Mih6)(h−s)+(Pi+1h−Mi+1h6)s, i=1,2,…,n−1,

where *P*_*si*_(*d*) is the segment of log-spline interpolation function, *P*_*s*_(*d*), for *d*_*i*_ ≤ *d* ≤ *d*_*i*+1_; *M*_*i*_ is second derivative at knot *x*_*i*_, *i* = 1, 2, …, *n*, which can be determined from the continuous condition of the first derivative for the cubic spline [19].

The interpolation procedure was accomplished by the “interp1” function of the Matlab package [19], as described by (5):

(5) $$\begin{array}{c} \text{Pc}=\text{interp}1\left(\ln d,P,\text{lndc},\text{“method”}\right), \end{array}$$

where ln⁡*d* and *P* are *n*-dimensional arrays of the logarithm of measured particle size and cumulative percentage, respectively; lndc is an array representing a desired classification of the logarithm of particle size; Pc is the interpolated cumulative percentage corresponding to lndc; and “method” specifies interpolation methods, where “linear,” “spline,” and “cubic” refer to piecewise linear interpolation, cubic spline interpolation, and monotone piecewise cubic interpolation, respectively.

### 2.2. Evaluation of the Log-Cubic, Log-Linear, and Log-Spline Methods

A leave-one-out cross-validation method was performed to assess the performance of the log-cubic, log-linear, and log-spline methods, using particle size data extracted from UNSODA database [18].

In the cross-validation procedure, data of one particle size were left out and interpolated with remaining data. Considering the boundary effect of interpolation, data of the first two and last two particle sizes were not left out in the validation process. Interpolated values were compared with omitted measured values to calculate the mean error (ME) and root mean square error (RMSE) with (6), which were then used to assess the performance of interpolation methods:

(6) $$\begin{array}{c} \text{ME}=\frac{1}{n-4}\sum_{i=3}^{n-2}\left(P_{i}-P_{i,i}\right),\\ \text{RMSE}={\left[\frac{1}{n-4}\sum_{i=3}^{n-2}{\left(P_{i}-P_{i,i}\right)}^{2}\right]}^{1/2}, \end{array}$$

where *n* is the number of particle size grade and *P*_*i*,*i*_ is the interpolated values of cumulative particle percentage.

Considering data requirement for interpolation and cross-validation, soils with five or more particle size grades  *n* ≥ 5 were considered. As a result, 394 soil samples were available from the UNSODA database [18] with the grade of particle size from 5 to 16, among which 94.2% has the grade number from 6 to 9.

**Figure 1:**
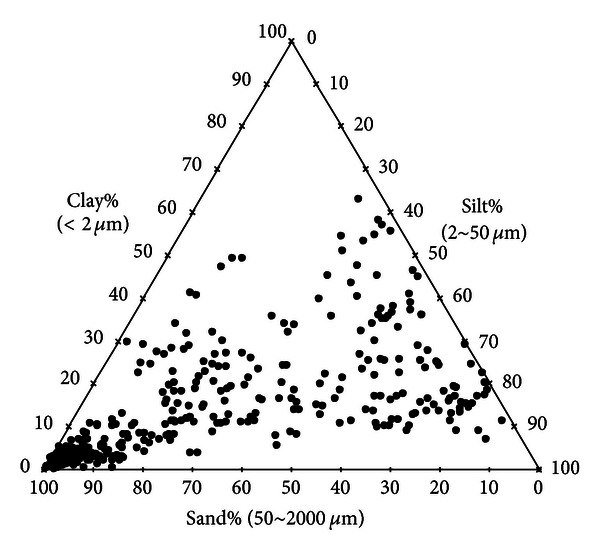
Textural composition of 353 soils samples used in the evaluation of interpolation methods.

Among these samples, particle size data of 353 samples covers the range from 2 *μ*m to 2000 *μ*m, which can be used to determine the clay (<2 *μ*m), silt (2~50 *μ*m), and sand (50~2000 *μ*m) fractions directly or through interpolation. The particle size distribution of these 353 soil samples covers a wide range of soil textures (Figure 1).

### 2.3. Generation of the PSD Curve

Once the interpolation method for the PSD curve was chosen, the PSD curve can be generated automatically from measured data, (*d*_*i*_, *P*_*i*_), *i* = 1, 2, …, *n*, with the following procedure.

(1) Determination of particle sizes for interpolation. Each particle size interval, [*di*, *d*_*i*+1_], *i* = 1, 2, …, *n* − 1, is divided into *m* subintervals with the endpoints defined in (7):
  (7) $$\begin{array}{c} d_{i,j}=\exp\left[\ln d_{i}+\frac{j\left(\ln d_{i+1}-\ln d_{i}\right)}{m}\right],\;j=1,2,\ldots ,m, \end{array}$$
  Consequently, *m*(*n* − 1) + 1 points are used for interpolation. To guarantee the smoothness of the PSD curve, *m* should not be less than 50, and *m* = 100 was used in this study.
(2) Interpolation of cumulative particle percentages (*P*_*i*,*j*_) corresponding to *d*_*i*,*j*_ from measured data using an appropriate interpolation method.
(3) Plot points for measured particle size analysis data, (*d*_*i*_, *P*_*i*_), *i* = 1, 2, …, *n*, in a semilogarithmic coordinates.
(4) Connect interpolated points, (*d*_*i*,*j*_, *P*_*i*,*j*_), *i* = 1, 2, …, *n* − 1, *j* = 1, 2, …, *m*, with line segments in succession in the same semilogarithmic coordinates.

## 3. Results and Discussion

### 3.1. Evaluation of Interpolation Methods

The monotonicity and the smoothness are essential requirements of PSD curves. From the principles of three interpolation methods, the log-linear method can guarantee the monotonicity of interpolated PSD curve, the log-spline method can guarantee the smoothness of interpolated PSD curve, while the log-cubic method can guarantee both the monotonicity and the smoothness of interpolated PSD curve. Therefore, the log-cubic method is essentially superior to log-linear and log-spline methods.

The log-linear, log-spline, and log-cubic methods were evaluated by the leave-one-out cross-validation method for 394 soil samples extracted from UNSODA database [18]. Average values of ME of these three methods are −0.4%, −0.6%, and −0.6%, respectively, which are all very close to the perfect value of 0. Average values of RMSE are 8.6%, 6.8%, and 6.3%, respectively, which indicates that the log-cubic method is superior to the other two methods in the average sense.

**Figure 2:**
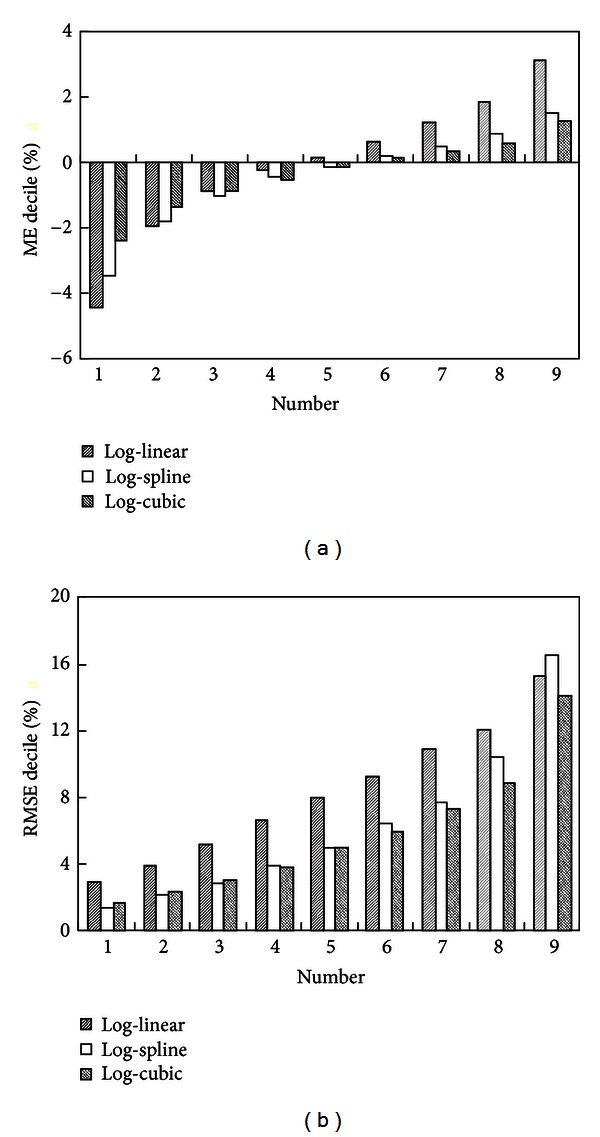
ME (a) and RMSE (b) deciles of the log-linear, log-spline, and log-cubic methods.

ME and RMSE deciles of these three methods are shown in Figure 2. The results show that most of the ME deciles of the log-cubic method are more close to the perfect value of 0 than those of log-linear and log-spline methods. As far as ME is concerned, the log-cubic method outperforms the log-linear and log-spline methods for 75% and 50% of soil samples, respectively. On the other hand, RMSE deciles of the log-cubic method are all less than those of the log-linear method and less than or slightly greater than those of the log-spline method. As far as RMSE is concerned, the log-cubic method outperforms the log-linear and log-spline methods for 95% and 84% soil samples, respectively. Therefore, the log-cubic method is superior to the log-linear and log-spline methods for interpolating PSDs.

Nemes et al. (1999) [15] used four procedures to estimate cumulative percentage at unmeasured particle size, including log-linear and spline interpolation methods, Gompertz curve regression method, and similarity method. Their results of cross-validation indicate that the similarity method is the most effective, which yielded the lowest RMSE ranging from 2% to 11% for different distances between particle size limits. RMSE value of the present log-cubic method is 6.3%, which corresponds to lower or medium RMSE values of the similarity method at smaller distances between particle size limits [15]. Moreover, the log-cubic method does not require a large external reference data set and can be used to estimate cumulative particle percentages at any size in the PSD range. The computation of the log-cubic method is much simpler than that of the similarity method. Therefore, the log-cubic method is appropriate to be used in the estimation of cumulative percentage at unmeasured particle size.

### 3.2. Comparison of Generated PSD Curves

**Figure 3:**
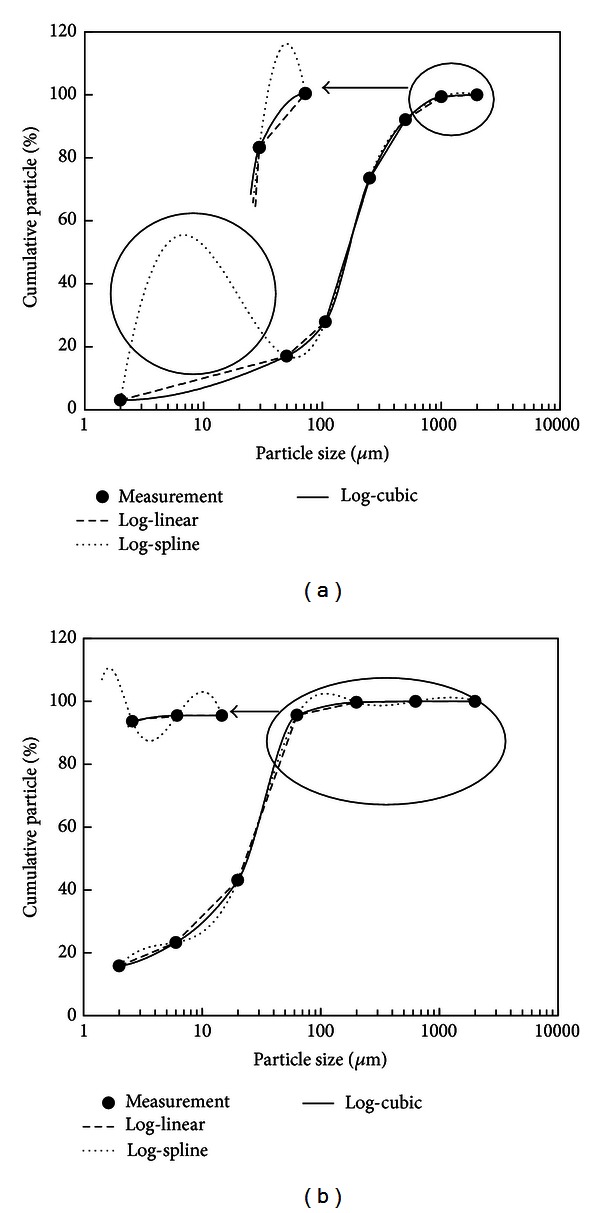
Examples of PSD curves generated from measured data (dots) using the log-linear (dashed lines), log-spline (dot lines), and log-cubic (solid lines) methods for soils nos. 1010 (a) and 1490 (b). Circles indicate particle size ranges in which PSD curves generated with the log-spline method are not monotonic.

Using the log-linear, log-spline, and log-cubic methods, cumulative particle percentages at unmeasured sizes can be estimated from measured data and used for the generation of PSD curve.  Figure 3 illustrates two PSD curves for two soil samples generated from 7 measured data. All generated PSD curves pass through measured data, which is an essential requirement of interpolation methods. For the log-linear method, the generated PSD curves are monotone but not smooth. For the log-spline method, the generated PSD curves are smooth but not monotone since an interpolated cubic spline is monotone only in specified conditions of measured data [17]. Impractical fluctuations in these PSD curves show that the log-spline method itself is not appropriate to generate a PSD curve, unless it is modified in some way [8]. While for the log-cubic method, the generated PSD curves are both smooth and monotone, which meet the essential requirements of a PSD curve. PSD curves of other soils generated with the log-cubic method all follow these essential requirements. These characters are the same as those of traditional freehand PSD curve, but the present result is objective and independent on persons involved.

Compared with other methods to generate a PSD curve, the log-cubic method is superior to the freehand method in its objectivity, superior to the log-linear method in the smoothness and accuracy of the interpolated PSD curve, and superior to the log-spline method in the monotonicity of the interpolated PSD curve. Therefore, the proposed log-cubic method provides an objective and reliable way to generate a PSD curve from limited soil particle analysis data with satisfactory precision.

### 3.3. Application of the Log-Cubic Method

The log-cubic method and the generated PSD curves can be used to estimate cumulative particle percentages at unmeasured sizes and to estimate characteristic particle sizes corresponding to specified cumulative particle percentages. These results can be further used in the conversion of different soil texture schemes, assessment of grading pattern, and estimation of soil hydraulic parameters and erodibility factor.

#### 3.3.1. Estimation of Cumulative Particle Percentage at Unmeasured Size

One direct use of the log-cubic method and the generated PSD curve is to estimate cumulative particle percentages at unmeasured sizes, which is necessary in the conversion of different soil texture schemes.

There are various classification schemes of soil particle size in different countries and different fields [1], such as schemes of the International Society of Soil Science (ISSS), U.S. Department of Agriculture (USDA). Incompatibility of different schemes may cause considerable confusions and inconveniences, and it is necessary to convert different classification schemes into the desired one [2, 15]. This conversion is essential in achieving compatibility among different classification schemes [15]. Besides, it is also useful in estimating soil hydraulic parameters and erodibility factor [8] from available pedotransfer functions when different classification schemes are used. For example, in the ROSETTA program for estimating soil hydraulic parameters with pedotransfer functions [3], soil hydraulic parameters were estimated from soil textural fractions based on the USDA scheme and other physical properties. When the ROSETTA program is used for soils with soil textural fractions of other classification schemes, conversion of the available scheme to the USDA scheme is required. 

The log-cubic method can be used in this conversion process, which was illustrated later with two soil samples shown in Figure 3.

For soil No. 1010 shown in Figure 3(a), clay (<2 *μ*m), silt (2~50 *μ*m), and sand (50~2000 *μ*m) fractions of the USDA scheme can be determined directly from measured data, which are 3%, 14%, and 83%, respectively. The soil texture is classified as loamy sand by the USDA scheme. However, the textural fractions are not available for the ISSS scheme, where the limit between silt and sand is 20 *μ*m. The cumulative particle percentage at 20 *μ*m can be estimated with the log-cubic method, which is 10.7%. Consequently, clay (<2 *μ*m), silt (2~20 *μ*m), and sand (20~2000 *μ*m) fractions of the ISSS scheme can be estimated to be 3%, 7.7%, and 89.3%, respectively, and the soil texture is classified as sand.

On the contrary, soil textural fractions of the ISSS scheme can be determined directly from measured data for soil no. 1490 (Figure 3(b)), which are 15.8%, 27.3%, and 56.9% for clay, silt, and sand particles, respectively. The soil texture is classified as sandy clay loam. The cumulative particle percentage at 50 *μ*m can be estimated with the log-cubic method, which is 90.0%. Then clay, silt, and sand fractions of the USDA scheme can be estimated to be 15.8%, 74.2%, and 10.0%, respectively, and the soil texture is classified as silt loam in the USDA scheme.

#### 3.3.2. Estimation of Characteristic Particle Sizes Corresponding to Specified Cumulative Particle Percentages

Characteristic particle sizes are sizes corresponding to specified cumulative particle percentages, such as *d*_10_, *d*_30_, *d*_50_, and *d*_60_ corresponding to cumulative particle percentages of 10, 30, 50, and 60, respectively. These characteristic particle sizes are essential data in the assessment of soil grading pattern with the uniformity index defined as *d*_60_/*d*_10_ and in the estimation of soil hydraulic properties from empirical or semiempirical formulae [23, 24]. However, these particle sizes cannot be obtained directly from particle size analysis results. They were usually determined graphically from the freehand PSD curve, which is subjective and error-prone. This problem can be solved by inverse interpolation of the PSD curve.

In the process to generate a PSD curve, dense points of particle size versus cumulative particle percentage were available using the log-cubic method, which are (*d*_*i*,*j*_, *P*_*i*,*j*_), *i* = 1, 2, …, *n* − 1,  *j* = 1, 2, …, *m*. These points can be used in the inverse interpolation to estimate characteristic particle sizes similar to (5), as described in (8):

(8) $$\begin{array}{c} \text{dc}=\exp\left(\text{interp}1\left(P,\ln d,\text{Pc},\text{“method”}\right)\right), \end{array}$$

where *P* and ln⁡*d* refer to *P*_*i*,*j*_ and ln⁡*d*_*i*,*j*_, *i* = 1, 2, …, *n* − 1,  *j* = 1, 2, …, *m*, respectively; Pc is the desired cumulative particle percentages, such as 10, 30, 50, and 60; dc is the interpolated characteristic particle sizes corresponding to Pc.

For example, *d*_10_, *d*_30_, *d*_50_, and *d*_60_ of soil no. 1010 shown in Figure 3(a) are estimated using the log-cubic interpolation method, which are 17.8 *μ*m, 114.0 *μ*m, 164.5 *μ*m, and 192.0 *μ*m, respectively. Then the uniformity index can be estimated to be 10.8, indicating a well-graded soil.

Two points need to be stressed about the previously mentioned inverse interpolation process. First, the desired cumulative particle percentage should be in the range of measured values, since the extrapolation results may be impractical. For example, *d*_10_ for the soil no. 1490 (Figure 3(b)) cannot be estimated satisfactorily using the previously mentioned inverse interpolation method or other similar methods. Second, values of cumulative particle percentage should be distinct, which is required by interpolation methods. For example, values of cumulative particle percentage for soil no. 1490 (Figure 3(b)) are kept constant (100) in the particle size range from 630 *μ*m to 2000 *μ*m, which should be omitted in the inverse interpolation. Using data from 2 *μ*m to 630 *μ*m, *d*_30_, *d*_50_, and *d*_60_ of this soil are estimated to be 10.3 *μ*m, 24.2 *μ*m, and 29.2 *μ*m, respectively.

Considering the satisfactory precision of the log-cubic method, this method provides an objective and reliable way for the conversion of different soil texture schemes and the estimation of characteristic particle sizes.

## 4. Conclusions

To overcome the subjectivity and uncertainty of the freehand PSD curve generated from limited data of particle size analysis, the log-cubic method was proposed for the interpolation of cumulative particle percentages at unmeasured sizes to generate the PSD curve automatically. The generated PSD curve passes through all measured data and is both smooth and monotone, which meets essential requirements of a PSD curve. Results of the leave-one-out cross-validation using 394 soil samples show that the interpolation precision of the log-cubic method is satisfactory compared with other methods. 

The proposed log-cubic method provides an objective and reliable way to generate a PSD curve from limited soil particle analysis data. This method and the generated PSD curve can be used to estimate cumulative particle percentages at unmeasured sizes and to estimate characteristic particle sizes corresponding to specified cumulative particle percentages, which can be further used in the conversion of different soil texture schemes, assessment of grading pattern, and estimation of soil hydraulic parameters and erodibility factor.
